# IgA and the gut-vagina axis

**DOI:** 10.3389/fimmu.2025.1547303

**Published:** 2025-04-17

**Authors:** Kazuhide Takada

**Affiliations:** ^1^ Division of Microbiology, Department of Pathology and Microbiology, Nihon University School of Medicine, Tokyo, Japan; ^2^ Division of Immune Homeostasis, Department of Pathology and Microbiology, Nihon University School of Medicine, Tokyo, Japan

**Keywords:** gut-vagina axis, microbiota, *Lactobacillus*, IgA, probiotics

## 1 Introduction

The microbiome in the reproductive tract influences both women’s and their offspring’s health (1–4). A highly diverse gut microbiota is considered healthy (5). However, the vaginal microbiota of a healthy reproductive-aged woman is often dominated by only one or two species of *Lactobacillus*, such as *L. crispatus*, *L. gasseri*, *L. iners*, and *L. jensenii* (6). Interestingly, this property is observed only in humans and not in other primates (7–9). Although some hypotheses have been proposed, the mechanism by which *Lactobacillus* dominates the human vaginal microbiota remains unknown (7, 10, 11).

Various treatments have been used to modify the vaginal microbiota to a *Lactobacillus*-dominant state against dysbiotic conditions. Probiotic treatment of the vaginal microbiota is promising (12). However, the indigenous vaginal microbiota frequently surpasses the colonization of the probiotic *L. crispatus* strain (CTV-05 strain) from the vaginal source (13). After 24 weeks, approximately 50% of patients who received this probiotic did not retain CTV-05 (12). Consequently, understanding the mechanism to regulate the vaginal microbiota by the host is crucial for developing novel therapies, including probiotics, to address conditions related to vaginal dysbiosis.

Immunoglobulins play important roles in regulating homeostasis and microbiota at mucosal sites. In the intestinal tract, immunoglobulin A (IgA) selectively attaches to microbes that have a close relationship with the host mucosa (14). IgA appears to serve a dual and context-sensitive function, acting to exclude pathogens while facilitating the colonization of beneficial commensals (14). Immunoglobulin G (IgG) plays a crucial role in promoting mucosal homeostasis in addition to regulating both non-invasive and invasive mucosal bacteria (15). In contrast to the gastrointestinal tract and other mucosal tissues, the antibodies found in the vagina are primarily IgG instead of IgA (15). In vagina, IgA, IgG, and IgM participate in immune defense (16). Although the function of vaginal IgG remains partially understood, it has been noted to capture viruses and protect the host against viral infections (17).

**Figure 1:**
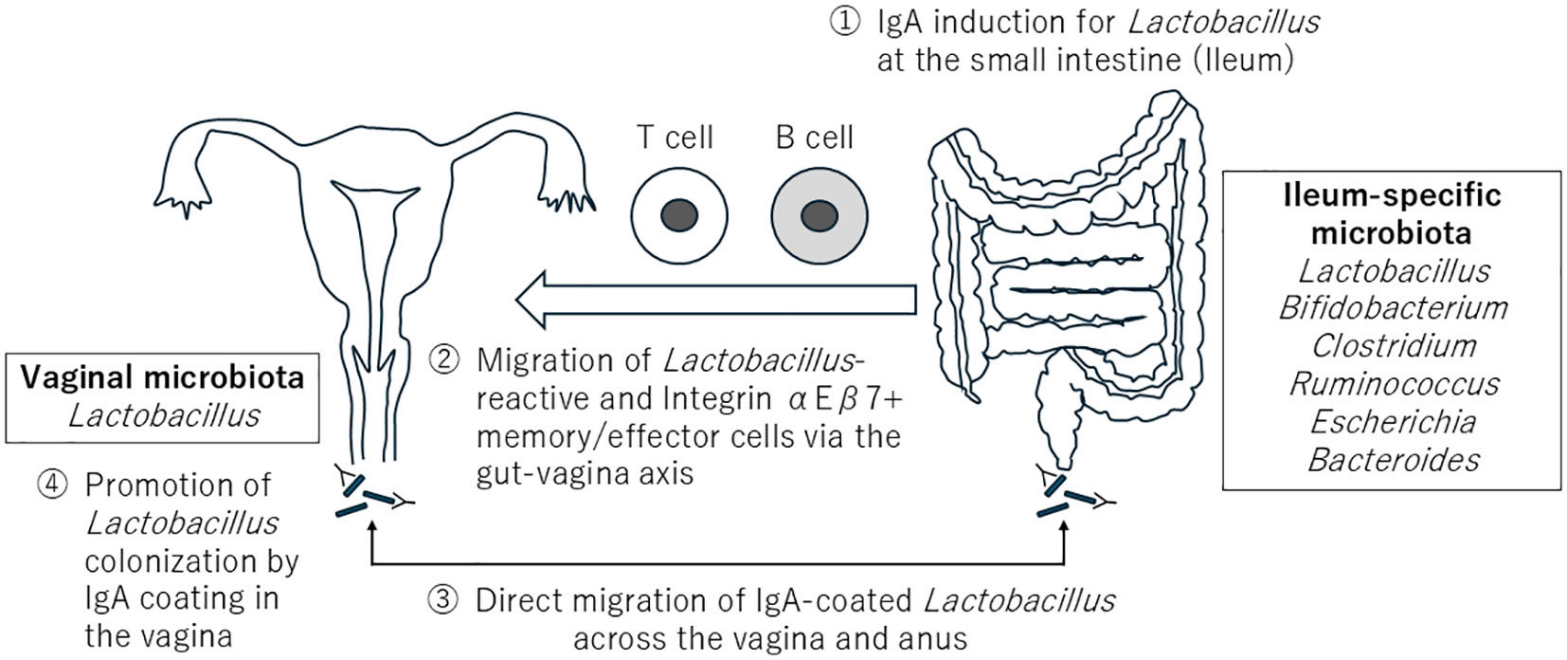
Hypothetical Schematic of colonization of the vagina by *Lactobacillus*. 1, 2: *Lactobacillus*-reactive and Integrin αEβ7+ memory/effector cells migrate from the small intestine to the vagina and produce *Lactobacillus*-specific IgA. 3: IgA-coated *Lactobacillus* may directly migrate from the gut to the vagina. 4: IgA-coated *Lactobacillus* promotes its stable colonization in the vagina.

Recent advances in IgA-seq have revealed that the prevalence of IgA-coated vaginal bacteria is elevated in *L. crispatus*-dominant microbiota compared to other microbiota compositions (18). Another study also reported that the levels of microbial IgA and IgG coating were lowest in individuals with diverse microbiota, particularly among women from ethnic minority groups (19). Accumulating evidence suggests that IgA-producing cells in the vagina originate from the intestine (20, 21). Furthermore, although IgG levels in cervicovaginal fluid were higher than those of IgA, it was IgA that predominantly coated the bacteria (22). Therefore, similar to the gastrointestinal tract, I hypothesize that IgA may regulate the composition of the vaginal microbiota in addition to its role in pathogen clearance. In this article, I discuss the possible regulation of the vaginal microbiota by the gut microbiota via IgA. Here, it is hypothesized that IgA induction for *Lactobacillus* occurs in the small intestine. Subsequently, *Lactobacillus*-reactive memory/effector cells migrate from the intestine to the vagina and produce *Lactobacillus*-specific IgA, increasing the number of IgA-coated *Lactobacillus*. Finally, these IgA-coated *Lactobacillus* strains promote stable vaginal colonization, highlighting IgA’s role in the gut-vagina axis (**Figure 1**).

### 1.1 Colonization of bacteria and IgA

Bacterial colonization in host organs is regulated by both direct and indirect mechanisms. Direct mechanisms include spatial nutrients or space competition, active antagonism, and metabolite inhibition (23). The direct mechanisms in the vagina are beyond the scope of this study and have been discussed elsewhere (11). Indirect mechanisms include mucus barrier function, oxygen limitation, and microbiota-mediated immune responses (23). In the gut, some bacterial symbionts stimulate the synthesis of polyreactive low-affinity IgA antibodies that may exhibit cross-reactivity with antigens from other bacterial species, which can affect the composition of the microbiota. Indeed, a significant percentage of commensal gut bacteria are coated with IgA antibodies (23).

Recently, IgA-seq has revealed that some microbes in the vagina are also coated with IgA and IgG (18, 22, 24). IgA and IgG are present in the cervicovaginal secretions bound to *L. crispatus*, *L. iners*, *Gardnerella vaginalis*, and *Prevotella bivia* (24). Interestingly, in a *L. crispatus*-dominant microbiota, the number of IgA-coated vaginal bacteria was found to be increased compared to other microbiota compositions (18). Therefore, IgA may regulate the vaginal microbiota, although whether it is polyreactive or species-specific remains unclear.

### 1.2 IgA induction for *Lactobacillus*

Next, where is IgA produced and how does it bind to *Lactobacillus*? In humans, IgA is classified into two subclasses, IgA1 and IgA2. The levels of IgA1 and IgA2 in the female genital tract secretions are approximately equal (25). These observed equal ratios of IgA1 and IgA2, coupled with the predominance of polymeric IgA in cervical secretions, indicates that IgA is synthesized locally in the mucosa (25). Notably, hysterectomy decreased immunoglobulin levels in the vagina, indicating that immunoglobulins generated locally and transferred from the bloodstream by uterine tissues, to some extent, contribute to humoral immunity in the vagina (26). While B cells represent a small cell population across all female reproductive tract tissues, plasma cells that produce IgA and IgG are primarily located in the cervix and, to a lesser degree, in the vagina (27). *In vivo*, initial infection with herpes simplex virus 2 (HSV2) fails to produce plasma cells within the lamina propria of the female reproductive tract. In contrast, upon secondary challenge with HSV2, circulating memory B cells that migrate into the female reproductive tract act as a source of rapid and substantial virus-specific IgG2b, IgG2c, and IgA secretion into the lumen of this tract (28). CD4 tissue-resident memory T cells generate interferon-γ, which results in the expression of chemokines like CXCL9 and CXCL10. Circulating memory B cells are attracted to the vaginal mucosa via a CXCR3-dependent mechanism, where they generate virus-specific IgG2b, IgG2c, and IgA, which are subsequently released into the lumen (28). However, these reactions have been observed under pathogenic conditions induced by HSV2. As vaginal *Lactobacillus* species are not pathogens, the mechanism of IgA induction for them under steady vaginal conditions remains obscure.

Another possible site for IgA induction for *Lactobacillus* is the gastrointestinal tract, which is the basis for the present hypothesis. Recent studies indicate that the primary origin of IgA targeting is the small intestine (14). Human gut-associated lymphoid tissue comprises multi-follicular Peyer’s patches (PP). Human PP consists of numerous individual follicles that extend throughout the entire length of the small intestine. Their density is increased in the terminal ileum, culminating in the formation of a lymphoid ring at the ileocecal junction (29). The PP serves as a location for adaptive immune priming and encompasses various specialized microanatomical niches that facilitate the effective initiation and propagation of immune responses (29). In humans, the core small intestinal microbiota includes *Streptococcus*, *Veillonella*, *Fusobacterium*, *Prevotella*, and *Haemophilus* (30). Notably, *Lactobacillus* is one of the segment-specific microbes found in the ileum (30). In mice, *Lactobacillus* is one of the dominant bacterial genus in the small intestine (31, 32). Therefore, IgA induction for *Lactobacillus* may occur mainly in the PP of the ileum in humans.

### 1.3 Migration (homing) of *Lactobacillus*-reactive immune cells

How do *Lactobacillus*-reactive immune cells migrate from the ileum to the female reproductive tract? Accumulating evidence from previous studies on orally administered human papillomavirus (HPV) vaccines based on genetically modified lactic acid bacteria provides us insights. *In vivo*, HPV16 L1-specific vaginal IgA was detected after oral administration of *L. lactis* transformed with two types of HPV16 L1-encoding plasmids (33). *L. lactis* with HPV16 L2-encoding plasmids also induces HPV16 L1-specific vaginal IgA in mice (34). In humans, oral administration of recombinant *L. lactis* expressing the HPV 16 E7 oncogene also induces the vaginal secretion of HPV 16 E7-specific IgA (20). α4β7 integrin -mediated homing to the intestine has already been established (35, 36), and a similar mechanism has been suggested for the vagina (21). These studies have indicated that the migration (homing) of *Lactobacillus*-reactive memory/effector cells occurs from the gut to the vagina (gut-vagina axis), and it is not surprising that antibodies recognizing the usual components of *Lactobacillus* (polyreactive or species-specific) are produced. To the best of my knowledge, no studies have reported that the same *Lactobacillu*s strains are shared between the intestine and vagina. Instead, phylogenetic studies of *L. crispatus* and *L. gasseri* in the gut and vagina suggest that different strains, adapted to their respective environments, may be established in the two organs, respectively (37, 38). Therefore, inducing *Lactobacillus*-specific rather than strain-specific antibodies would be important.

Overall, IgA induction for *Lactobacillus* can occur in the small intestine (especially in the ileum). Then *Lactobacillus*-reactive and integrin αEβ7+ memory/effector cells migrate from the intestine to the vagina and produce *Lactobacillus*-specific IgA, resulting in the increased number of IgA-coated *Lactobacillus* in the vagina. Another possibility to increase the number of IgA-coated *Lactobacillus* in the vagina is through direct migration from the gut. IgA-coated *L. jensenii* has been confirmed in fecal samples from healthy women (39). Additionally, probiotic *Lactobacillus* strains administered orally were confirmed in the vagina (40–43). Therefore, these observations suggest that *Lactobacillus* already coated with IgA in the gastrointestinal tract may also migrate directly to the vagina.

Finally, these IgA coatings may promote stable colonization of *Lactobacillus* in the vagina (**Figure 1**). *In vitro* study suggested that IgA can enhance the mucosal binding of *Lactobacillus* in the gut (14). Therefore, IgA-coated *Lactobacillus* adhering to vaginal mucus may facilitate their vaginal colonization; however, further studies are needed to determine the effects of differences in the composition of mucus between the intestinal tract and vagina. Notably, a lower quantity of IgA attachment per bacterium was observed when women with *L. crispatus*-dominant microbiota exhibited a higher level of IgA coating on vaginal bacteria than those with other microbiota compositions (18). Another investigation revealed a notable preference for IgA coating of taxa linked to vaginal dysbioses (bacterial vaginosis), such as *Sneathia* and *Prevotella* species (22). Hence, an unknown mechanism that distinguishes the degree of IgA coating may balance pathogen clearance and host-microbial symbiosis in the vagina. Interestingly, *Lactobacillus* obtained from undernourished diet-fed mice demonstrated a significantly reduced capacity to bind IgA, indicating that *Lactobacillus* may have adapted mechanisms for evading IgA (14). Therefore, the nutritional environment may also regulate the IgA coating of *Lactobacillus* in the vagina.

## 2 Discussion

Mutualistic symbiosis has developed as a result of millions of years of coevolution between the host and microorganisms, wherein the microbiota supports host metabolic processes and the host gives bacteria nourishment and a preferable environment (23). The dominance of the human vagina by *Lactobacillus* can also be regarded as a result of its coevolution with humans (11), which contribute to women’s health (1–3). Nonetheless, the process by which *Lactobacillus* establishes dominance in the vaginal microbiota is still not fully understood.

Notably, among *Lactobacillus* species, only *L. iners* is regarded as an undesirable bacteria because it is associated with recurrent bacterial vaginosis (dysbiosis of the vaginal microbiota) (44). The significance of *L. iners* during the coevolution between humans and *Lactobacillus* remains unknown. However, the metabolic capacity and small genome size of *L. iners* compared to other vaginal *Lactobacillus* species indicate the potential for targeting metabolic differences (such as cysteine and oleic acid) to either inhibit *L. iners* or even enhance other species of *Lactobacillus* (45, 46). These metabolic differences may provide clues to coevolution.

In addition to the present hypothesis involving IgA, another mechanism of the gut-vagina axis is the estrobolome (47) which was not the focus of the present study. Briefly, some bacteria in the gastrointestinal tract can deconjugate estrogens that were previously conjugated in the liver. Subsequently, the reabsorption of deconjugated estrogen into the systemic circulation occurs. Circulating estrogen affects the distal epithelium of the vagina by modifying the physiological characteristics of vaginal epithelial cells, including glycogen and mucus production. Elevated glycogen levels promote the dominance of *Lactobacillus* in the vaginal environment because glycogen acts as a crucial energy source for vaginal *Lactobacillus* (48–50).

In conclusion, IgA induction for *Lactobacillus* in the small intestine may promote colonization of this bacterium in the vagina via IgA regulation. If the present hypothesis is valid, prior or simultaneous oral administration of probiotics could enhance the colonization of the same bacteria administered vaginally. A further understanding of the relationship between the female reproductive tract and other organs is required to establish effective treatments.

## References

[1] France M Alizadeh M Brown S Ma B Ravel J. Towards a deeper understanding of the vaginal microbiota. Nat Microbiol. (2022) 7:367–78. doi: 10.1038/s41564-022-01083-2 PMC8910585 35246662

[2] Kaluanga Bwanga P Tremblay-Lemoine PL Timmermans M Ravet S Munaut C Nisolle M. The endometrial microbiota: challenges and prospects. Medicina (Kaunas). (2023) 59(9):1540. doi: 10.3390/medicina59091540 37763663 PMC10534531

[3] Gao S Wang J. Maternal and infant microbiome: next-generation indicators and targets for intergenerational health and nutrition care. Protein Cell. (2023) 14(11):807–23. doi: 10.1093/procel/pwad029 PMC10636639 37184065

[4] Abramov VM Kosarev IV Priputnevich TV Machulin AV Abashina TN Chikileva IO. S-layer protein 2 of vaginal Lactobacillus crispatus 2029 enhances growth, differentiation, VEGF production and barrier functions in intestinal epithelial cell line Caco-2. Int J Biol Macromol. (2021) 189:410–9. doi: 10.1016/j.ijbiomac.2021.08.150 34437917

[5] Kriss M Hazleton KZ Nusbacher NM Martin CG Lozupone CA. Low diversity gut microbiota dysbiosis: drivers, functional implications and recovery. Curr Opin Microbiol. (2018) 44:34–40. doi: 10.1016/j.mib.2018.07.003 30036705 PMC6435260

[6] Ravel J Gajer P Abdo Z Schneider GM Koenig SS McCulle SL. Vaginal microbiome of reproductive-age women. Proc Natl Acad Sci United States America. (2011) 108 Suppl 1:4680–7. doi: 10.1073/pnas.1002611107 PMC3063603 20534435

[7] Stumpf RM Wilson BA Rivera A Yildirim S Yeoman CJ Polk JD. The primate vaginal microbiome: comparative context and implications for human health and disease. Am J Phys Anthropol. (2013) 152 Suppl 57:119–34. doi: 10.1002/ajpa.22395 24166771

[8] Yildirim S Yeoman CJ Janga SC Thomas SM Ho M Leigh SR. Primate vaginal microbiomes exhibit species specificity without universal Lactobacillus dominance. ISME J. (2014) 8:2431–44. doi: 10.1038/ismej.2014.90 PMC4260710 25036926

[9] Chen Z Yeoh YK Hui M Wong PY Chan MCW Ip M. Diversity of macaque microbiota compared to the human counterparts. Sci Rep. (2018) 8:15573. doi: 10.1038/s41598-018-33950-6 30349024 PMC6197227

[10] Miller EA Beasley DE Dunn RR Archie EA. Lactobacilli dominance and vaginal pH: why is the human vaginal microbiome unique? Front Microbiol. (2016) 7:1936. doi: 10.3389/fmicb.2016.01936 28008325 PMC5143676

[11] Hayashida S Takada K Melnikov VG Komine-Aizawa S Tsuji NM Hayakawa S. How were Lactobacillus species selected as single dominant species in the human vaginal microbiota? Coevolution of humans and Lactobacillus. Med Hypotheses. (2022) 163:110858. doi: 10.1016/j.mehy.2022.110858

[12] Cohen CR Wierzbicki MR French AL Morris S Newmann S Reno H. Randomized trial of lactin-V to prevent recurrence of bacterial vaginosis. New Engl J Med. (2020) 382:1906–15. doi: 10.1056/NEJMoa1915254 PMC7362958 32402161

[13] Antonio MA Meyn LA Murray PJ Busse B Hillier SL. Vaginal colonization by probiotic Lactobacillus crispatus CTV-05 is decreased by sexual activity and endogenous Lactobacilli. J Infect Dis. (2009) 199:1506–13. doi: 10.1086/598686 19331578

[14] Huus KE Bauer KC Brown EM Bozorgmehr T Woodward SE Serapio-Palacios A. Commensal bacteria modulate immunoglobulin A binding in response to host nutrition. Cell Host Microbe. (2020) 27:909–21.e5. doi: 10.1016/j.chom.2020.03.012 32289261

[15] Chen K Magri G Grasset EK Cerutti A. Rethinking mucosal antibody responses: IgM, IgG and IgD join IgA. Nat Rev Immunol. (2020) 20:427–41. doi: 10.1038/s41577-019-0261-1 PMC10262260 32015473

[16] Zhou JZ Way SS Chen K. Immunology of the uterine and vaginal mucosae. Trends Immunol. (2018) 39:302–14. doi: 10.1016/j.it.2018.01.007 29433961

[17] Kwon MS Lee HK. Host and microbiome interplay shapes the vaginal microenvironment. Front Immunol. (2022) 13:919728. doi: 10.3389/fimmu.2022.919728 35837395 PMC9273862

[18] Breedveld AC Schuster HJ van Houdt R Painter RC Mebius RE van der Veer C. Enhanced IgA coating of bacteria in women with Lactobacillus crispatus-dominated vaginal microbiota. Microbiome. (2022) 10:15. doi: 10.1186/s40168-021-01198-4 35074009 PMC8787895

[19] Schuster HJ Breedveld AC Matamoros SPF van Eekelen R Painter RC Kok M. The interrelation between microbial immunoglobulin coating, vaginal microbiota, ethnicity, and preterm birth. Microbiome. (2024) 12:99. doi: 10.1186/s40168-024-01787-z 38802950 PMC11131309

[20] Mohseni AH Taghinezhad SS Keyvani H. The first clinical use of a recombinant Lactococcus lactis expressing human papillomavirus type 16 E7 oncogene oral vaccine: A phase I safety and immunogenicity trial in healthy women volunteers. Mol Cancer Ther. (2020) 19:717–27. doi: 10.1158/1535-7163.MCT-19-0375 31645442

[21] Kobayashi O Taguchi A Nakajima T Ikeda Y Saito K Kawana K. Immunotherapy that leverages HPV-specific immune responses for precancer lesions of cervical cancer. Taiwanese J Obstet Gynecol. (2024) 63:22–8. doi: 10.1016/j.tjog.2023.10.002 38216264

[22] Murphy K Gromisch M Srinivasan S Wang T Wood L Proll S. IgA coating of vaginal bacteria is reduced in the setting of bacterial vaginosis (BV) and preferentially targets BV-associated species. Infect Immun. (2024) 92:e0037323. doi: 10.1128/iai.00373-23 38099624 PMC10790818

[23] Caballero-Flores G Pickard JM Núñez G. Microbiota-mediated colonization resistance: mechanisms and regulation. Nat Rev Microbiol. (2023) 21:347–60. doi: 10.1038/s41579-022-00833-7 PMC10249723 36539611

[24] Liu R Pollock J Huibner S Udayakumar S Irungu E Ngurukiri P. Microbe-binding antibodies in the female genital tract: associations with the vaginal microbiome and genital immunology. J Immunol (Baltimore Md: 1950). (2024) 213:1516–27. doi: 10.4049/jimmunol.2400233 39345194

[25] Mestecky J Fultz PN. Mucosal immune system of the human genital tract. J Infect Dis. (1999) 179 Suppl 3:S470–4. doi: 10.1086/jid.1999.179.issue-s3 10099122

[26] Mestecky J Russell MW. Induction of mucosal immune responses in the human genital tract. FEMS Immunol Med Microbiol. (2000) 27:351–5. doi: 10.1111/j.1574-695X.2000.tb01449.x 10727891

[27] Wira CR Rodriguez-Garcia M Patel MV. The role of sex hormones in immune protection of the female reproductive tract. Nat Rev Immunol. (2015) 15:217–30. doi: 10.1038/nri3819 PMC4716657 25743222

[28] Oh JE Iijima N Song E Lu P Klein J Jiang R. Migrant memory B cells secrete luminal antibody in the vagina. Nature. (2019) 571:122–6. doi: 10.1038/s41586-019-1285-1 PMC6609483 31189952

[29] Mörbe UM Jørgensen PB Fenton TM von Burg N Riis LB Spencer J. Human gut-associated lymphoid tissues (GALT); diversity, structure, and function. Mucosal Immunol. (2021) 14:793–802. doi: 10.1038/s41385-021-00389-4 33753873

[30] Yersin S Vonaesch P. Small intestinal microbiota: from taxonomic composition to metabolism. Trends Microbiol. (2024) 32:970–83. doi: 10.1016/j.tim.2024.02.013 38503579

[31] Peng Y Wei J Jia X Luan F Man M Ma X. Changes in the microbiota in different intestinal segments of mice with sepsis. Front Cell Infect Microbiol. (2022) 12:954347. doi: 10.3389/fcimb.2022.954347 36704101 PMC9871835

[32] Hugenholtz F de Vos WM. Mouse models for human intestinal microbiota research: a critical evaluation. Cell Mol Life Sci: CMLS. (2018) 75:149–60. doi: 10.1007/s00018-017-2693-8 PMC5752736 29124307

[33] Cho HJ Shin HJ Han IK Jung WW Kim YB Sul D. Induction of mucosal and systemic immune responses following oral immunization of mice with Lactococcus lactis expressing human papillomavirus type 16 L1. Vaccine. (2007) 25:8049–57. doi: 10.1016/j.vaccine.2007.09.024 17936447

[34] Yoon SW Lee TY Kim SJ Lee IH Sung MH Park JS. Oral administration of HPV-16 L2 displayed on Lactobacillus casei induces systematic and mucosal cross-neutralizing effects in Balb/c mice. Vaccine. (2012) 30:3286–94. doi: 10.1016/j.vaccine.2012.03.009 22426329

[35] Shouval DS. α4β7 expression guides B cells to front lines of defense in the gut. Mucosal Immunol. (2022) 15:192–4. doi: 10.1038/s41385-021-00476-6 34931001

[36] Mora JR von Andrian UH. Differentiation and homing of IgA-secreting cells. Mucosal Immunol. (2008) 1:96–109. doi: 10.1038/mi.2007.14 19079167

[37] Zhang Q Zhang L Ross P Zhao J Zhang H Chen W. Comparative genomics of lactobacillus crispatus from the gut and vagina reveals genetic diversity and lifestyle adaptation. Genes. (2020) 11(4):360. doi: 10.3390/genes11040360 32230824 PMC7230607

[38] Pan M Hidalgo-Cantabrana C Goh YJ Sanozky-Dawes R Barrangou R. Comparative Analysis of Lactobacillus gasseri and Lactobacillus crispatus Isolated From Human Urogenital and Gastrointestinal Tracts. Front Microbiol. (2019) 10:3146. doi: 10.3389/fmicb.2019.03146 32038579 PMC6988505

[39] Sun J Qi C Zhu H Zhou Q Xiao H Le G. IgA-targeted lactobacillus jensenii modulated gut barrier and microbiota in high-fat diet-fed mice. Front Microbiol. (2019) 10:1179. doi: 10.3389/fmicb.2019.01179 31178854 PMC6542990

[40] Reid G Bruce AW Fraser N Heinemann C Owen J Henning B. Oral probiotics can resolve urogenital infections. FEMS Immunol Med Microbiol. (2001) 30:49–52. doi: 10.1111/j.1574-695X.2001.tb01549.x 11172991

[41] Morelli L Zonenenschain D Del Piano M Cognein P. Utilization of the intestinal tract as a delivery system for urogenital probiotics. J Clin Gastroenterol. (2004) 38:S107–10. doi: 10.1097/01.mcg.0000128938.32835.98 15220672

[42] Strus M Chmielarczyk A Kochan P Adamski P Chelmicki Z Chelmicki A. Studies on the effects of probiotic Lactobacillus mixture given orally on vaginal and rectal colonization and on parameters of vaginal health in women with intermediate vaginal flora. Eur J Obstet Gynecol Reprod Biol. (2012) 163:210–5. doi: 10.1016/j.ejogrb.2012.05.001 22721635

[43] Russo R Edu A De Seta F. Study on the effects of an oral lactobacilli and lactoferrin complex in women with intermediate vaginal microbiota. Arch Gynecol Obstet. (2018) 298:139–45. doi: 10.1007/s00404-018-4771-z 29637269

[44] Petrova MI Reid G Vaneechoutte M Lebeer S. Lactobacillus iners: friend or foe? Trends Microbiol. (2017) 25:182–91. doi: 10.1016/j.tim.2016.11.007 27914761

[45] Bloom SM Mafunda NA Woolston BM Hayward MR Frempong JF Abai AB. Cysteine dependence of Lactobacillus iners is a potential therapeutic target for vaginal microbiota modulation. Nat Microbiol. (2022) 7:434–50. doi: 10.1038/s41564-022-01070-7 PMC10473153 35241796

[46] Zhu M Frank MW Radka CD Jeanfavre S Xu J Tse MW. Vaginal Lactobacillus fatty acid response mechanisms reveal a metabolite-targeted strategy for bacterial vaginosis treatment. Cell. (2024) 187:5413–30.e29. doi: 10.1016/j.cell.2024.07.029 39163861 PMC11429459

[47] Takada K Melnikov VG Kobayashi R Komine-Aizawa S Tsuji NM Hayakawa S. Female reproductive tract-organ axes. Front Immunol. (2023) 14:1110001. doi: 10.3389/fimmu.2023.1110001 36798125 PMC9927230

[48] Łaniewski P Ilhan ZE Herbst-Kralovetz MM. The microbiome and gynaecological cancer development, prevention and therapy. Nat Rev Urol. (2020) 17:232–50. doi: 10.1038/s41585-020-0286-z PMC9977514 32071434

[49] Łaniewski P Herbst-Kralovetz MM. Connecting microbiome and menopause for healthy ageing. Nat Microbiol. (2022) 7:354–8. doi: 10.1038/s41564-022-01071-6 PMC9977513 35246661

[50] Baker JM Al-Nakkash L Herbst-Kralovetz MM. Estrogen–gut microbiome axis: physiological and clinical implications. Maturitas. (2017) 103:45–53. doi: 10.1016/j.maturitas.2017.06.025 28778332

